# Protein phosphatase 1 regulatory subunit 18 suppresses the transcriptional activity of NFATc1 via regulation of c-fos

**DOI:** 10.1016/j.bonr.2021.101114

**Published:** 2021-08-04

**Authors:** Kazuma Yasuda, Takuma Matsubara, Tomohiko Shirakawa, Tatsuo Kawamoto, Shoichiro Kokabu

**Affiliations:** a Division of Molecular Signaling and Biochemistry, Department of Health Improvement, Kyushu Dental University, 2-6-1 Manazuru, Kokurakita-ku, Kitakyushu, Fukuoka 803-8580, Japan; b Division of Orofacial Functions and Orthodontics, Department of Health Improvement, Kyushu Dental University, 2-6-1 Manazuru, Kokurakita-ku, Kitakyushu, Fukuoka 803-8580, Japan

**Keywords:** PPP1r18, protein phosphatase 1 regulatory subunit 18, NFATc1, nuclear factor of activated T cells 1, M-CSF, macrophage colony stimulating factor, RANKL, receptor activator nuclear factor kappa B ligand, RANK, receptor activator nuclear factor kappa B, TRAP, tartrate resistant acid phosphatase, c-fos, Fos proto-oncogene, AP-1 transcription factor subunit, c-Jun, Jun proto-oncogene, AP-1 transcription factor subunit, Dc-stamp, dendrocyte expressed seven transmembrane protein, Ctsk, cathepsin K, Src, Rous sarcoma oncogene, PP1, protein phosphatase 1, GapDH, glyceraldehyde-3-phosphate dehydrogenase, Osteoclast, PPP1r18, NFATc1, c-Fos

## Abstract

The transcription factor NFATc1 and its binding partner AP-1 (a complex containing c-fos and c-Jun) play a central role in osteoclast differentiation. NFATc1 and AP-1 promote the expression of target genes such as *Acp5, Ctsk* and also auto-regulate *NFATc1* expression as well. We previously reported that protein phosphatase 1 regulatory subunit 18 (PPP1r18) is a negative regulator of osteoclast bone resorption by inhibiting cell attachment to bone matrix. We also reported that PPP1r18 potentially regulates NFATc1 expression during osteoclast differentiation. To further explore this, in this study we have examined the effect of PPP1r18 on NFATc1 expression and activity by overexpressing PPP1r18 during the early stage of osteoclast differentiation. We found that PPP1r18 suppressed NFATc1 expression through inhibition of the transcriptional activity of NFATc1. Since PPP1r18 does not regulate NFATc1 directly, we next explored the involvement of AP-1. Our data showed that c-fos phosphorylation and nuclear localization were reduced by PPP1r18 overexpression. Further experiments showed that overexpression of c-fos together with PPP1r18 rescued NFATc1 expression and transcriptional activity. Moreover, c-fos activity inhibition by PPP1r18 was canceled by mutation of the phosphatase binding site of PPP1r18. Taken together, PPP1r18-regulated phosphatase activity targets c-fos phosphorylation and suppresses subsequent NFATc1 expression and activity.

## Highlights

• PPP1r18 suppresses osteoclast differentiation.
• PPP1r18 suppresses c-fos phosphorylation and nuclear localization.
• PPP1r18 suppresses NFAT via c-fos.

## 1 Introduction

The transcription factor NFATc1 is essential for RANKL-induced osteoclast differentiation (Takayanagi et al., 2002; Ikeda et al., 2004; Asagiri et al., 2005). Osteoclasts do not form in NFATc1 deficient mice (Asagiri et al., 2005). Furthermore, NFATc1 overexpression in the absence of RANKL stimulation is sufficient to induce osteoclast differentiation from osteoclast precursor cells (Takayanagi et al., 2002). Upon activation, NFATc1 is dephosphorylated and moves from the cytosol into the nucleus (Sato et al., 2006). Nuclear NFATc1 activates transcription of target genes such as dendrocyte expressed seven transmembrane protein (*Dc-stamp*), acid phosphatase 5, tartrate resistant (*Acp5* / *Trap*) and cathepsin *K* (*Ctsk)* to promote osteoclast differentiation) (Ikeda et al., 2004; Asagiri et al., 2005; Takayanagi, 2007; Miyamoto, 2011). Interestingly, NFATc1 not only induce these downstream genes, but also up-regulates expression of NFATc1 itself (Asagiri et al., 2005).

NFATc1 transcription activity is increased by binding of NFATc1 with the AP-1 complex consisting of c-fos and c-Jun (Takayanagi, 2007; Teitelbaum, 2004). Both c-fos and c-Jun are activated by RANKL signaling and are also necessary for NFATc1 stimulation of osteoclast differentiation (Takayanagi et al., 2002; Ikeda et al., 2004; Matsuo et al., 2004). c-Fos deficient mice exhibit severe osteopetrosis because of defective osteoclast differentiation (Johnson et al., 1992; Asagiri and Takayanagi, 2007). NFATc1 expression is also decreased in c-fos deficient osteoclast precursors after RANKL stimulation most likely due to defective NFATc1 auto-regulation (Takayanagi et al., 2002). Furthermore, c-fos expression accelerates NFATc1 transcriptional activity at the TRAP promoter (Takayanagi et al., 2002). Although the mechanisms regulating c-fos are not fully understood, C-terminal phosphorylation of c-fos enhances protein stability, nuclear localization and transcriptional activity. However, proteins that regulate c-fos phosphorylation in osteoclast precursors are not well understood (Chen et al., 1996; Tanos et al., 2005). c-Jun is also essential for osteoclastogenesis and transgenic mice expressing dominant negative c-Jun also show osteopetrosis and reduction of osteoclast formation (Ikeda et al., 2004). Dominant negative c-Jun can inhibit complex formation with c-fos and can suppress NFATc1 transcription activity on the TRAP promoter (Ikeda et al., 2004). c-Jun is phosphorylated by c-Jun NH2-terminal kinase (JNK), a member of the mitogen-activated protein kinase (MAPK) family. Downstream of RANKL signaling, phosphorylated c-JUN binds to c-fos and translocates into the nucleus (Ikeda et al., 2004).

Differentiated osteoclasts strongly attach to bone matrix via the activity of a Src tyrosine kinase after which they secrete protons and matrix metalloproteases such as Cathepsin K to resorb bone matrix (Takayanagi, 2007; Soriano et al., 1991; Boyce et al., 1992; Suda et al., 1997; Suda et al., 1999; Horne et al., 2005; Takahashi et al., 2007; Baron and Kneissel, 2013). We recently identified a phosphatase regulating protein, PPP1r18, as a Src binding protein (Matsubara et al., 2018a). PPP1r18 is a protein phosphatase 1 (PP1) binding protein, and is also reported to be involved in the organization of actin filaments () and the regulation of c-Jun activity in osteoblasts (Addison et al., 2019). We also reported that the expression of PPP1r18 was decreased during osteoclast differentiation and overexpression of PPP1r18 in differentiated osteoclasts disturbed actin ring formation and suppressed bone resorption (Matsubara et al., 2018a). In addition to this, we also showed that NFATc1 mRNA was increased in PPP1r18 knockdown mature osteoclasts. This suggests that PPP1r18 also regulates osteoclast differentiation via NFATc1 transcription control. Here in this study, we have now examined this possibility and determined the role of PPP1r18 on NFATc1 during osteoclastogenesis.

## 2 Materials and methods

### 2.1 Cell culture

RAW264.7 cells were obtained from KAC Co., ltd. (Kyoto, Japan), HEK293 cells were obtained from the RIKEN Cell Bank (Ibaraki, Japan) and Cos 7 cells were obtained from the National Institutes of Biomedical Innovation, Health and Nutrition (Osaka, Japan). Cells were cultured in Dulbecco's Modified Eagle Medium (DMEM) (Fujifilm Wako, Osaka, Japan) supplemented with 10% (*v*/v) fetal bovine serum (FBS) (Sigma-Aldrich, St. Louis, MO) and 1% (v/v) penicillin/streptomycin (Thermo Fisher Scientific, San Jose, CA).

### 2.2 Reagents and antibodies

Soluble RANKL (sRANKL) was obtained from the Oriental Yeast Company, Ltd. (Shiga, Japan). Anti-PPP1r18 antibody (sc-376,816), anti-c-fos antibody (sc-52), anti-c-Jun antibody (sc-74,543), anti-IκBα antibody (sc-371) and anti-NFATc1 antibody (sc-7294) were obtained from Santa Cruz biotechnology, Inc. (Santa Cruz, TX). Anti-phospho c-Jun (#2361) and horseradish peroxidase (HRP)-conjugated anti-Histone H3(3H1) antibody (#5192) were obtained from Cell Signaling Technology (Danvers, MA), and anti-β-actin (A5441) antibody were obtained from Sigma-Aldrich. anti-c-Fos (phospho Ser362, GTX3291) was obtained from GeneTex Inc. (Irvine, CA). HRP-conjugated anti-mouse, anti-rabbit and anti-Rat IgG secondary antibodies were obtained from Jackson immunoresearch laboratories, Inc. (West Grove, PA). Anti-myc-tag antibody and HRP-conjugated anti-Glyceraldehyde-3-phosphate Dehydrogenase (GapDH) antibody were obtained from Medical and biological laboratories co. ltd. (Nagoya, Japan).

### 2.3 Plasmids and constructs

PPP1r18 plasmid was purchased from GE Healthcare and subcloned into myc-tagged vector as previously described (Matsubara et al., 2018a). Human c-fos plasmid was purchased from Applied Biological Materials (Richmond, BC, Canada) and then subcloned into pcDNA3.1 (−) vector (Thermo Fisher Scientific) with a c-terminal flag-tag (5′-gattacaaggacgacgatgacaagtag-3′). NFATc1 and constitutively activated NFATc1 were obtained from addgene (Watertown, MA). pShuttle-CMV-lacZ vector was purchased from Agilent technologies (Santa Clara, CA). pGL4.30[luc2P/NFAT-RE/Hygro] Vector and pRL-TK vector were obtained from Promega corporation (Madison, WI).

### 2.4 Adenovirus production

Empty, wild type PPP1r18 (PPP1r18 WT), PPP1r18 mutated Ile540 and Phe542 to Gly (PPP1r18 IGFG), control shRNA and PPP1r18 shRNA adenovirus were produced as previously described (Matsubara et al., 2018a). Flag tagged c-fos was digested with *Pme*I (New England Biolabs) and inserted into cosmid vector pAxEFwtit2 (Takara bio Inc.). Cosmid vectors were digested with BspT104i (Takara bio Inc.) and transfected into HEK293 cells with Lipofectamine 2000 (Thermo Fisher Scientific) to obtain primary adenovirus. Fresh adenovirus was produced by infection of HEK293 cells with this adenovirus. Adenovirus titers were determined by the modified point assay as described before (Ikeda et al., 2004; Matsubara et al., 2018a).

### 2.5 Determination of DNA introduction efficiency

5000 cells of RAW 264.7 cells were plated at 96 well plate and infected empty or GFP adenovirus (M.O.I. = 50) and culture for 2 days. 10,000 cells of Cos 7 cells were plated at 96 well plate and transfected empty or GFP vectors by Lipofectamine 2000 and culture for 2 days. GFP were observed with BZ-X810. Infection or transfection efficiency was calculated of the ratio GFP positive cell number vs total cell number. The infection efficiency into RAW 264.7 cells was about 90% (Fig. S1). The transfection efficiency was almost 80% (Fig. S2).

### 2.6 Determination of cell viability

Empty adenovirus was infected at RAW 264.7 cells for 6 h. After infection, 5000 cells of adenovirus infected or non-infected RAW 264.7 cells were plated at 96 well plate. The cell viability was determined by Cell Counting Kit-8 (Dojindo, Kumamoto, Japan) by manufacturer protocol (Ogawa et al., 2019). Cell viability was not affected by adenovirus infection (Fig. S3).

### 2.7 Phagocytosis assay

Empty adenovirus was infected at RAW 264.7 cells for 6 h. After infection, 5000 cells of adenovirus infected or non-infected RAW 264.7 cells were plated at 96 well plate for 1 day. The phagocytosis was determined with phagocytosis assay kit (Cayman chemical, Ann Arbor, MI). In brief, latex beads -rabbit IgG-FITC complex was add the culture medium at 1:200 dilution and incubated for 2 h at 37 °C. Cells were washed with assay buffer and stained with Hoechst 33342 (Dojindo). The cells were observed with BZ-X810 (Keyence, Osaka, Japan). Phagocytosis of RAW 264.7 cells was not affected by adenovirus infection (Fig. S4).

### 2.8 Osteoclast differentiation and tartrate-resistant acid phosphatase (TRAP) staining

RAW 264.7 cells were cultured (10,000 cells/cm^2^) in αMEM supplemented with 10% FBS, 1% penicillin/streptomycin and 100 ng/ml sRANKL for 4 days, with media changes every 2 days. Differentiated cells were fixed with 10% formaldehyde for 10 min, ethanol-acetone (1:1) mixture for 1 min, and then washed with distilled water. Fixed cells were stained for 30 min with a TRAP solution containing Fast Red violet LB salt, naphthol-MX phosphate, and *N*,*N*-dimethylformamide in 50 mM acetate buffer as described before (Matsubara et al., 2018a; Matsubara et al., 2017; Matsubara et al., 2020). TRAP-positive multinuclear (> 3 nuclei) cells (TRAP (+) MNCs) were regarded as osteoclasts. Osteoclast differentiation from RAW 264.7 cells was not affected by adenovirus infection (Fig. S5).

### 2.9 Retrovirus production

PPP1r18-myc was subcloned to pQCIXN vector (Takara bio) with PCR methods using PrimSTAR Max DNA polymerase (Takara bio). Control or hPPP1r18-myc vector were transfected in Platinum-E Retroviral Packaging Cell Line, Ecotropic (Plat-E) cells (Cell Biolabs, Inc., CA) and incubated for 1 day. The supernatant of the retrovirus production cells was moved to RetroNectin coated dishes and incubated for 6 h to make retrovirus coating dish.

### 2.10 Osteoclast differentiation from mouse bone marrow cells

Bone marrow cells were harvested from 5 weeks old C57BL/6 N mice and incubated for 3 h. Non-adherent cells were corrected and cultured with 100 ng/μl M-CSF (Fujifilm Wako) for 3 days to differentiate to bone marrow macrophages (BMMs). The BMMs were plated at 100,000 cells/cm^2^ on retrovirus coating dish and cultured with 20 ng/μl M-CSF and 100 ng/μl RANKL for 5 days. The differentiated osteoclasts were determined by TRAP staining.

### 2.11 Western blotting analysis

For whole cell lysates, cells were solubilized with lysis buffer containing 1% Triton X, centrifuged and supernatants collected as described before (Matsubara et al., 2018a). For cytosolic and nuclear fractionation, cells were lysed with hypotonic buffer (20 mM 2-[4-(2-hydroxyethyl)piperazin-1-yl]ethanesulfonic acid (HEPES), 10 mM KCl, 1 mM ethylenediaminetetraacetic acid (EDTA), 0.2% NP-40, 10% Glycerol, 1.5 mM MgCl2, 1 mM Na3VO4, 1 mM Phenylmethylsulfonyl fluoride (PMSF), 10 μg/ml Aprotinin, 10 μg/ml Leupeptin and 1 mM Dithiothreitol (DTT)), centrifuged at 15,000 ×*g* for 20 min at 4 °C and the supernatant collected as the cytosol fraction. Remaining precipitates were then solubilized in hypertonic buffer (20 mM HEPES, 10 mM KCl, 0.2 mM EDTA, 20% Glycerol, 400 mM NaCl, 1.5 mM MgCl2, 1 mM Na3VO4, 1 mM PMSF, 10 μg/ml Aprotinin, 10 μg/ml Leupeptin and 1 mM DTT) with vortexing. After centrifugation at 15,000 ×*g* for 15 min at 4 °C, the supernatants were collected as the nuclear extract. Protein fractions were then boiled for 5 min at 95 °C with sample buffer as described before (Matsubara et al., 2018a). The samples were separated by SDS-polyacrylamide gel electrophoresis and transferred to a nitrocellulose membrane. The membrane was incubated with blocking buffer (5% bovine serum albumin, 250 mM Tris, 26.8 mM KCl, 1.37 M NaCl, 0.1% Tween 20 and 0.01 M sodium azide adjusted to pH 7.4 with HCl), corresponding primary antibodies and HRP-conjugated secondary antibodies. Blots were developed with an Immobilon Western chemiluminescent HRP substrate (Merck Millipore) and recorded with MultiImager II purchased from BioTools Inc. (Gunma, Japan).

### 2.12 Real-time quantitative PCR

Total RNA of osteoclasts was isolated using FastGene™ RNA Basic Kit from Nippon genetics Co., ltd. (Tokyo, Japan). cDNA was synthesized from 1 μg of total RNA with High-Capacity cDNA Reverse Transcription Kit (Thermo fisher Scientific). Real-time quantitative PCR was performed using, PowerUp SYBR™ Green Master Mix from Thermo Fisher Scientific and the primers indicated below in Table 1 in a QuantStudio 3 Real-time PCR system (Thermo Fisher Scientific). All reactions were performed in triplicate and analyzed. Expression levels were normalized by Gapdh expression (Matsubara et al., 2020; Matsubara et al., 2018b; Matsubara et al., 2018c; Inoue et al., 2020).

**Table 1:** qPCR primers.

Gene	Forward primer (5′ to 3′)	Reverse primer (5′ to 3′)
*Acp5*	tcctggctcaaaaagcagtt	acatagcccacaccgttctc
*Ctsk*	gggaagcaagcactggataa	ccgagccaagagagcatatc
*c-FOS (human)*	gcctctcttactaccactcacc	agatggcagtgaccgtgggaat
*GapDH*	aactttggcattgtggaagg	acacattggggtaggaaca
*Nfatc1*	ggtgctgtctggccataact	gcggaaaggtggtatctcaa
*PPP1r18*	acagagtggaggctgaagtc	tcaccggaacaatccctgaa

.

### 2.13 Luciferase assay

Cos7 cells were plated in 96 well plates and transfected with NFATc1, PPP1r18, c-fos, pGL4.30[luc2P/NFAT-RE/Hygro] and pRL-TK with Lipofectamine 2000. After 24 h, the cells were washed with PBS and lysed with passive lysis buffer contained in Dual-Luciferase Reporter Assay kit purchased from Promega corporation. Luciferase activity was measured with Luciferase Reporter Assay kit and normalized by the renilla thymidine kinase activity (Kokabu et al., 2017).

### 2.14 Data analysis and statistics

Independent experiments were performed at least three times. Differences between groups were analyzed by the Student's *t*-test or one-way ANOVA analysis to determine statistical significance. A value of *P* < 0.05 was considered statistically significant. Data are expressed as mean values ± standard deviation of means (mean ± SD; n = number of experiments).

## 3 Results

### 3.1 PPP1r18 represses the transactivation of NFATc1 and suppresses osteoclastogenesis

**Fig. 1:**
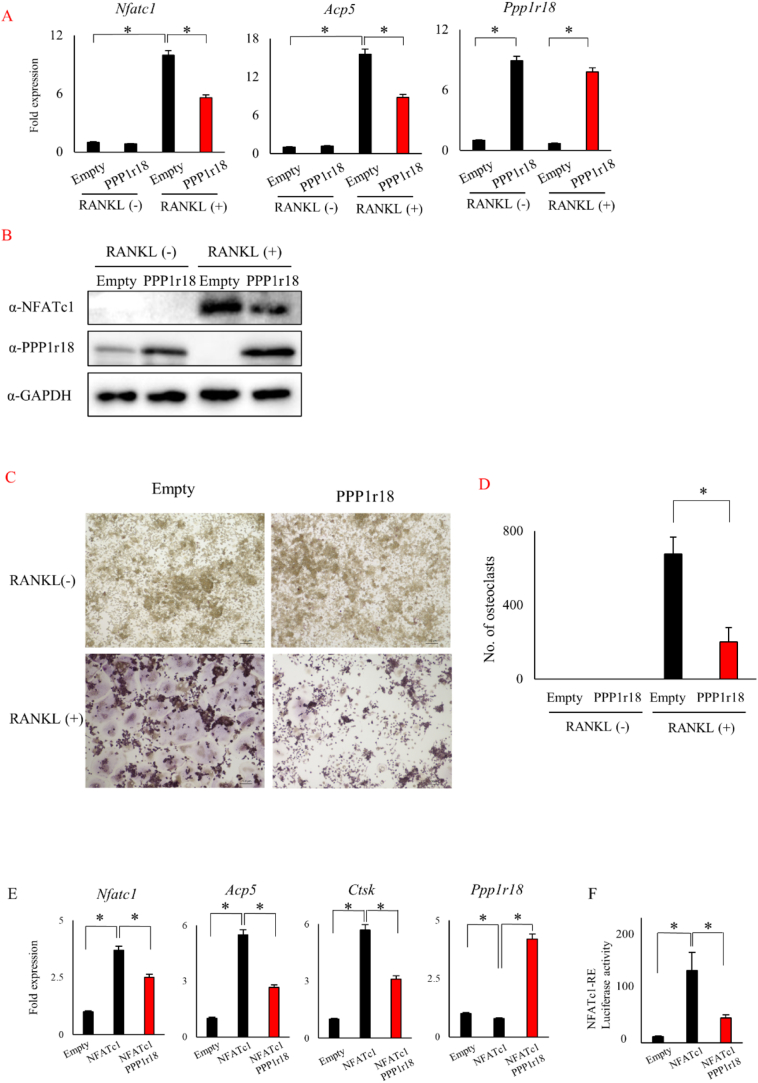
PPP1r18 decreases NFATc1 expression and suppresses osteoclast differentiation. (A) Empty vector or PPP1r18 vector were introduced into RAW 264.7 cells by adenovirus. After 2 days, RAW 264.7 cells were stimulated with or without 100 ng/ml sRANKL for 48 h. mRNA was harvested and expression level of mRNA were determined by qPCR as described in materials and methods (mean ± SD; *n* = 3). *, *p* < 0.05. (B - D) Empty, PPP1r18 adenovirus was infected into RAW 264.7 cells. After 2 days incubation, RAW 264.7 cells were cultured with or without 100 ng/ml sRANKL for 5 days. (B) Cells were lysed at or day 5 and the expression level of NFATc1 and PPP1r18 determined by western blotting analysis. (C) After 5 days culture, cells were fixed and stained by TRAP staining. Scale bar, 100 μm. (D) Number of TRAP positive multinuclear cells were counted (mean ± SD; *n* = 3). *, *p* < 0.05. (E) Empty, NFATc1, or NFATc1 with PPP1r18 adenovirus were introduced into RAW 264.7 cells with adenovirus for 2 days. mRNAs were harvested and expression level of mRNA were determined by qPCR (mean ± SD; *n* = 3). *, *p* < 0.05. (F) Empty, NFATc1, or NFATc1 with PPP1r18 plasmids were transfected with pGL4.30[luc2P/NFAT-RE/Hygro] along with pRL-TK vectors in Cos 7 cells. After 24 h culture, luciferase activity was measured and normalized by renilla (mean ± SD; n = 3). *, p < 0.05.

We firstly confirmed and examined whether PPP1r18 is involved in NFATc1 expression and/or early stage osteoclast differentiation. *Nfatc1* mRNA expression that is increased by RANKL stimulation was decreased after overexpression of PPP1r18 (Fig. 1A). mRNA levels of *Acp5*, a direct target gene of *NFATc1*, were also less expressed after PPP1r18 overexpression (Fig. 1A). Consistent with this, both NFATc1 protein levels and osteoclast differentiation induced by RANKL stimulation was suppressed by PPP1r18 overexpression (Fig. 1B, C, D, S6). On the other hand, NFATc1 expression and osteoclast differentiation were increased after PPP1r18 knock down (Fig. S7).

We next examined the effect of PPP1r18 on the transcriptional activity of NFATc1 induced by the overexpression of NFATc1 since NFATc1 has been shown to up-regulate transcription of NFATc1 (Ikeda et al., 2004; Asagiri et al., 2005; Takayanagi, 2007; Miyamoto, 2011). Over-expression of PPP1r18 suppressed mRNA levels of *Nfatc1*, *Acp5* and *Ctsk* induced by NFATc1 (Fig. 1E). PPP1r18 also repressed the activity of a luciferase reporter containing NFATc1 response elements from the Interleukin 2 promoter (Fig. 1F). These results suggest that PPP1r18 represses transactivation of NFATc1 and that PPP1r18 decreases the mRNA levels of NFATc1 target genes including NFATc1 itself, and thereby suppresses osteoclast differentiation.

### 3.2 PPP1r18 suppresses NFATc1 and osteoclast differentiation via phosphatase activity

**Fig. 2:**
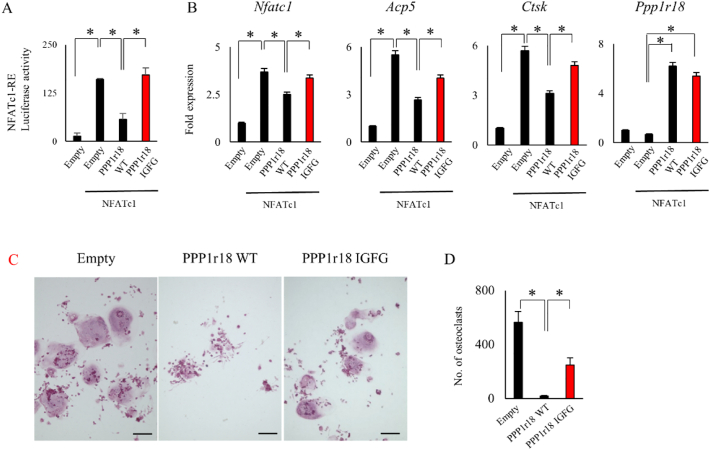
Phosphatase activity is required for the repression of NFATc1 by PPP1r18. (A) Empty, PPP1r18 wild type (PPP1r18 WT), or defective PP1-binding mutant of PPP1r18 (PPP1r18 IGFG) with or without NFATc1 plasmids were transfected along with pGL4.30[luc2P/NFAT-RE/Hygro] and pRL-TK vectors in Cos 7 cells. After 24 h culture, luciferase activity was measured and normalized by renilla (mean ± SD; *n* = 3). *, *p* < 0.05. (B) Empty, PPP1r18 WT or PPP1r18 IGFG, with or without NFATc1 were infected into RAW 264.7 cells by adenovirus. After 2 days incubation, RAW 264.7 cells were lysed for mRNA harvesting. Indicated mRNA expression was examined by qPCR (mean ± SD; *n* = 3). *, *p* < 0.05. (C and D) Empty, PPP1r18 WT, or PPP1r18 IGFG adenovirus were infected into RAW 264.7 cells. After 2 days incubation, RAW 264.7 cells were cultured with 100 ng/ml RANKL for 5 days. After fixation, cells were stained by TRAP staining. Scale bar, 100 μm. (C) Number of TRAP positive multinuclear cells were counted (mean ± SD; n = 3). *, p < 0.05. (D).

PPP1r18 binds PP1 and regulates its phosphatase activity. PPP1r18 also negatively regulates osteoclastic bone resorption (Matsubara et al., 2018a; Kao et al., 2007). We next examined whether PPP1r18 requires PP1 phosphatase activity for repression and suppression of NFATc1 transcriptional activity and osteoclast differentiation. We used a PPP1r18 mutant (PPP1r18 IGFG) that cannot bind PP1. PPP1r18 IGFG failed to repress the transcriptional activity of NFATc1 (Fig. 2A) and only mildly reduced NFATc1 target gene mRNA expression (Fig. 2B). In addition to this, PPP1r18 IGFG slightly suppressed osteoclast formation (Fig. 2C, D), suggesting that PP1 and phosphatase activity are required for the regulation of NFATc1 transcriptional activity and osteoclast differentiation by PPP1r18.

### 3.3 PPP1r18 does not affect the phosphorylation status of NFATc1

**Fig. 3:**
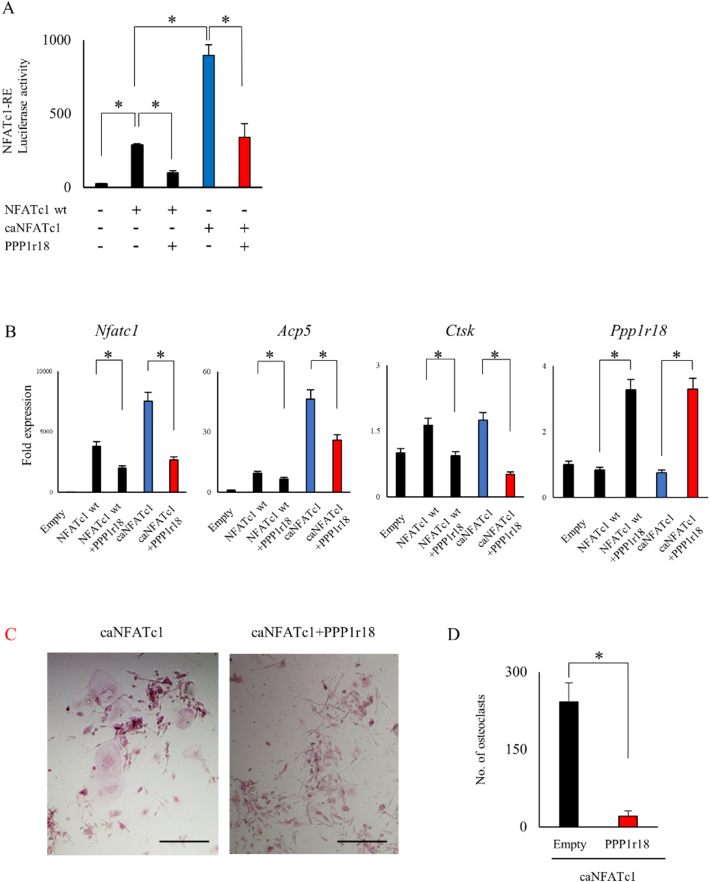
Suppression of constitutively activate NFATc1 by PPP1r18. (A) Empty, NFATc1 wild type (WT) or constitutively activated (ca) NFATc1 with or without PPP1r18 plasmids were transfected along with pGL4.30[luc2P/NFAT-RE/Hygro] and pRL-TK vectors in Cos 7 cells. After 24 h culture, luciferase activity was measured and normalized by renilla (mean ± SD; *n* = 4). *, *p* < 0.05. (B) Empty, NFATc1 WT or caNFATc1 with or without PPP1r18 were infected into RAW 264.7 cells by adenovirus. After 2 days incubation, RAW 264.7 cells were lysed for mRNA harvesting. Indicated mRNA expression was examined by qPCR (mean ± SD; *n* = 3). *, p < 0.05. (C and D) caNFATc1 and PPP1r18 WT adenovirus were introduced into RAW 264.7 cells and cultured for 3 days. After fixation, cells were stained by TRAP staining. Scale bar, 100 μm. (C) Number of TRAP positive multinuclear cells were counted (mean ± SD; n = 3). *, p < 0.05. (D).

Upon activation, NFATc1 is de-phosphorylated in the cytoplasm and subsequently translocated into the nucleus (Takayanagi, 2007). To examine whether PPP1r18 is involved in the phosphorylation status of NFATc1, we used a constitutively activated form of NFATc1 (caNFATc1) (Monticelli and Rao, 2002). Over expression of caNFATc1 resulted in higher luciferase activity than NFATc1 wild type (wt) (Fig. 3A). PPP1r18 not only reduced the luciferase activity induced by NFATc1 wt but also that by caNFATc1 (Fig. 3A). Corresponding with this, PPP1r18 overexpression suppressed *Acp5* and *Ctsk* mRNA expression induced by caNFATc1 expression (Fig. 3B). Furthermore, the number of osteoclasts induced by caNFATc1 overexpression was reduced by PPP1r18 overexpression (Fig. 3C and D). These results suggest that PPP1r18 represses the transactivation of NFATc1 without affecting the phosphorylation status of NFATc1, and so consequently negatively regulates osteoclast differentiation.

### 3.4 PPP1r18 regulates the phosphorylation and nuclei translocation of c-fos

**Fig. 4:**
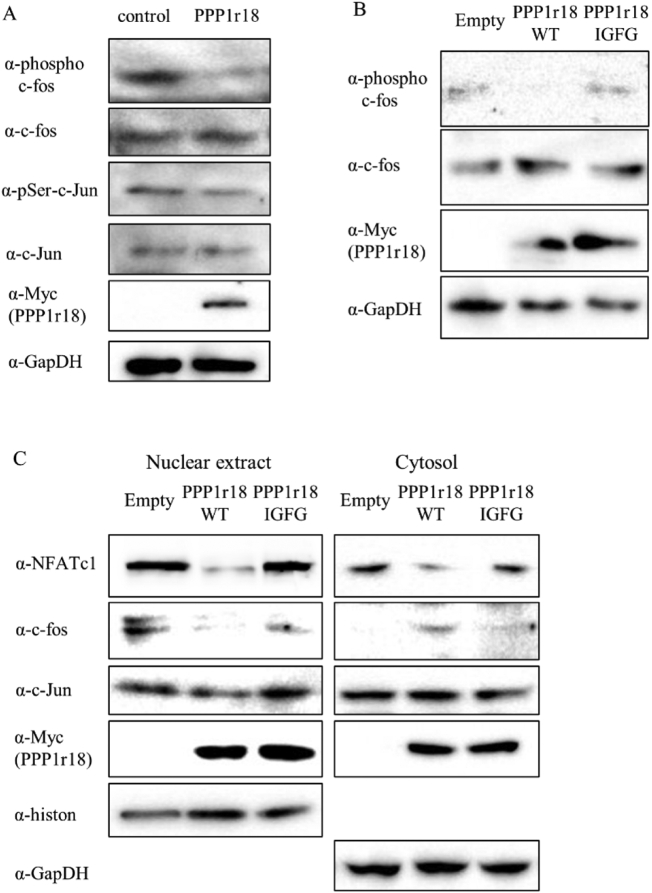
PPP1r18 alters c-fos phosphorylation and localization. (A) RAW 264.7 cells were infected with empty or PPP1r18 adenovirus. After 2 days incubation, RAW 264.7 cells were cultured with 100 ng/ml sRANKL for 1 h. Whole cell lysates were harvested. The indicated protein levels were examined by western blotting analysis. (B) Empty, wild type PPP1r18 (PPP1r18 WT), or defective PP1-binding mutant of PPP1r18 (PPP1r18 IGFG) adenovirus were infected into RAW 264.7 cells. After 2 days incubation, RAW 264.7 cells were cultured with 100 ng/ml sRANKL for 1 h. Cell were lysed and examined the expression level of indicated proteins by western blotting analysis. (C) Empty, PPP1r18 WT or PPP1r18 IGFG adenovirus were infected into RAW 264.7 cells. After 2 days incubation, RAW 264.7 cells were cultured with 100 ng/ml sRANKL for 2 days. Nuclear extracts or whole cell lysates were harvested. The indicated protein expression levels were examined by western blotting analysis.

To address how PPP1r18 suppresses the transcriptional activity of NFATc1, we focused on the coactivating NFATc1-binding partners c-fos and c-Jun (Ikeda et al., 2004; Takayanagi, 2007; Teitelbaum, 2004). Phosphorylation of c-fos and c-Jun by MAPKs after RANKL stimulation is essential for transcriptional activity of the AP-1 complex (Teitelbaum, 2004; Tanos et al., 2005). We therefore examined the effect of PPP1r18 on the phosphorylation status of c-fos and c-Jun. Overexpression of PPP1r18 reduced c-fos phosphorylation following RANKL treatment in both RAW 264.7 cells and BMMs (Fig. 4A, S8). However, PPP1r18 did not change the levels of phosphorylated c-Jun (Fig. 4A). The reduction in phosphorylated c-fos did not occur following overexpression of the PPP1r18 IGFG mutant, which does not possess phosphatase-binding capacity (Fig.4B). We next examined the localization of c-fos and c-Jun since phosphorylation is essential for their nuclear translocation (Teitelbaum, 2004; Chen et al., 1996; Tanos et al., 2005). Corresponding with total protein level, overexpression of PPP1r18 reduced the NFATc1 in nuclei. Overexpression of PPP1r18 did not change the protein level of c-Jun in both the nuclear fraction and the whole cell lysate. However, the levels of c-fos in the nuclear fraction was decreased after PPP1r18 overexpression despite no change in the total protein level of c-fos (Fig. 4C). These results suggest that PPP1r18 suppresses the phosphorylation and nuclear translocation of c-fos thereby disturbing NFATc1/c-fos complex formation at the promotor region of NFATc1 target genes.

### 3.5 PPP1r18 does not target c-fos to suppress NFATc1 and osteoclast differentiation

**Fig. 5:**
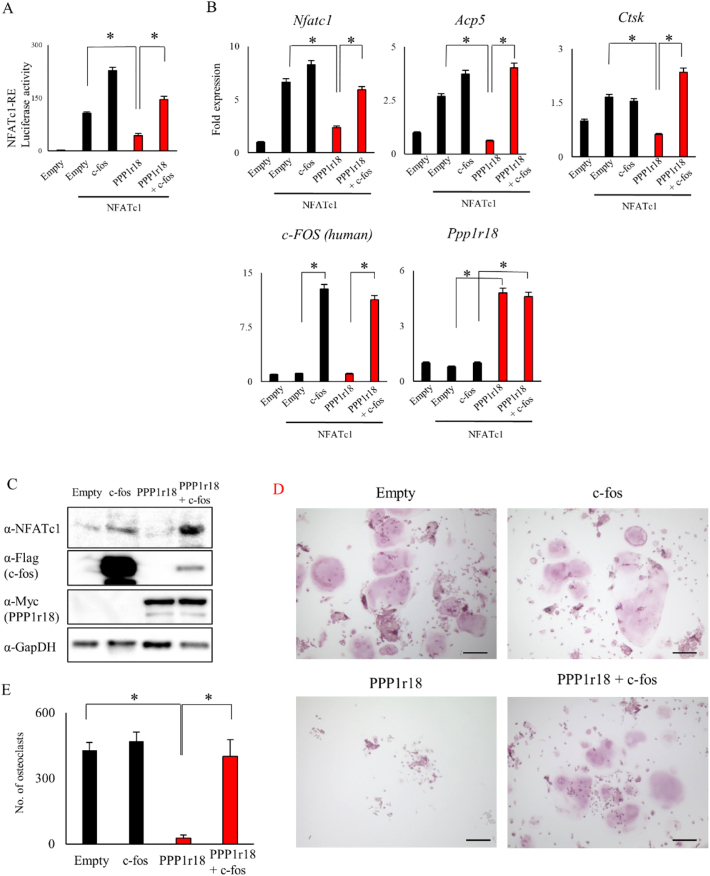
Over-expression of c-fos rescues the suppressive effect of PPP1r18 on transactivation of NFATc1 during osteoclast differentiation. (A) Empty, NFATc1, PPP1r18, and/or c-fos plasmids were transfected with pGL4.30[luc2P/NFAT-RE/Hygro] along with pRL-TK vectors in Cos 7 cells indicating the combination. After 24 h culture, luciferase activity was measured and normalized by renilla (mean ± SD; *n* = 3). *, *p* < 0.05. (B) Empty, NFATc1, PPP1r18, and/or c-fos were infected into RAW 264.7 cells by adenovirus system indicating the combination. After 2 days incubation, mRNA was harvested and mRNA levels determined by real-time PCR (mean ± SD; n = 3). *, p < 0.05. (C - E) Empty, c-fos, PPP1r18, or PPP1r18 with c-fos adenovirus were infected into RAW 264.7 cells. After 1 day incubation, RAW 264.7 cells were cultured with 100 ng/ml RANKL for 5 days. (C) Cell were lysed and the expression level of indicated protein examined by western blotting analysis. (D) After fixation, cells were stained by TRAP staining. Scale bar, 100 μm. (E) Number of TRAP positive multinuclear cells were counted (mean ± SD; n = 3 wells). *, p < 0.05.

Finally, we examined whether PPP1r18 targets c-fos by attempting to rescue the phenotypic effects of PPP1r18 by overexpression of human c-fos. The introduction of c-fos canceled the suppressive effect of PPP1r18 on the transactivation of NFATc1 (Fig. 5A). Consistent with this, c-fos also rescued the suppressive effect of PPP1r18 on NFATc1 target genes such as *Nfatc1*, *Acp5*, or *Ctsk* (Fig. 5B). In addition, overexpression of c-fos also canceled the suppressive effect of PPP1r18 on NFATc1 and osteoclastogenesis following RANKL stimulation (Fig. 5C - 5E). These results suggest that c-fos could completely cancel the suppressive effect of PPP1r18 on the transactivation of NFATc1 and osteoclastogenesis because PPP1r18 de-phosphorylates a protein upstream of c-fos, which then changes the phosphorylation status of c-fos.

## 4 Discussion

In this paper, we identified PPP1r18 as a new repressor of NFATc1. Briefly, PPP1r18 and the PP1 complex de-phosphorylate c-fos and disturb the formation of a complex between AP-1 and NFATc1. This represses the transcription of target genes (*Nfatc1, Acp5, Ctsk*) and consequently suppresses the initiation of osteoclast differentiation downstream of RANKL. In mature osteoclasts, PPP1r18 and the PP1 complex also inhibit Src function and actin ring formation by altering Src localization and suppressing Src's binding to actin regulatory proteins (Matsubara et al., 2018a). Combining the findings of these studies suggest that PPP1r18 regulates PP1 activity and negatively regulates osteoclast differentiation and function during both initiation and maturation.

We found that PPP1r18 and the PP1 complex suppressed c-fos phosphorylation. Phosphorylation of Ser362 at the c-fos c-terminus by MAPK is required for c-fos stabilization and nuclear translocation in mesenchymal cell lines (Tanos et al., 2005; Okazaki and Sagata, 1995). Similar to this, c-fos phosphorylation is probably important for stabilization and nuclear localization in osteoclast precursors. If c-fos is directory dephosphorylated by PPP1r18, c-fos should be inactivated when c-fos was over expressed. However, the overexpression of c-fos canceled the suppressive effect of PPP1r18 on c-fos, NFATc1 and osteoclast differentiation. In addition, we could not detect direct interaction of PPP1r18 and c-fos by immunoprecipitation (data not shown). These phenomena suggest that PPP1r18 indirectly regulates the phosphorylation of c-fos downstream of p38 MAPK signaling and/or phosphatidylinositol 3-kinase (PI3K)/Akt signaling which are known to regulate phosphorylation of c-fos (Tanos et al., 2005; Li et al., 2017). Further investigation is needed to identify the target of PPP1r18 and the c-fos regulator protein. Compared with c-fos, phosphorylation and localization of c-Jun, another component of the AP-1 complex was not altered by PPP1r18 overexpression. c-Jun expression also did not affect NFATc1 activity in NFATc1 RE luciferase activity (data not shown). These data suggests that c-Jun is not the target of PPP1r18 in osteoclast.

Previous and present our study indicates that PPP1r18 is physiological negative regulator in osteoblastogenesis because knockdown of PPP1r18 increases osteoclast differentiation and bone resorbing activity and have a negative feedback mechanism that the expression of PPP1r18 is decreased during osteoclast differentiation (Matsubara et al., 2018a). In contrast, PPP1r18 promotes osteoblast differentiation by promoting the binding of c-Jun and Nascent polypeptide-associated complex and co-regulator α (NACA) for transcription of Type I collagen, osteocalcin and other osteoblast differentiation gene (Addison et al., 2019; Akhouayri et al., 2005; Akhouayri and St-Arnaud, 2007; Meury et al., 2010; Pellicelli et al., 1861). In addition, the expression of PPP1r18 is higher in osteoblast than in fibroblast (NCBI Gene Expression Omnibus (GEO) data set GDS2091) (supple Fig. 9). Thus, PPP1r18 may work as bone remodeling factor by not only suppressing osteoblastogenesis but also accelerating osteoblastogenesis physiologically.

NFATc1 and the AP-1 complex play a central role not only in osteoclast differentiation but also in the differentiation and activation of lymph cells (Rao et al., 1997; Peng et al., 2001; Macian, 2005; Mauri and Bosma, 2012). NFATc1 and c-fos are expressed and activated by immunoreceptors and promote T and B cell differentiation in immune responses and inflammation (Peng et al., 2001; Macian, 2005). PPP1r18 expression is higher in immune tissues and cells such as bone marrow, spleen and lymph nodes (Lin et al., 2010; Lin et al., 2011). These results suggest that PPP1r18 may function to suppress NFATc1 in immune cells. Rheumatoid arthritis (RA) and osteoarthritis (OA) are major bone disease and regarded as auto immune disease. In these diseases, activated immune system cells secreted inflammatory cytokine such as tumor necrosis factor alpha (TNF-α) and RANKL. These cytokines promote osteoclastogenesis, in consequence, causes the bone destruction. Ciclosporin, the calcineurin - NFATc1 inhibitor is also effective as a treatment of RA and OA. Therefore, there is the possibility that PPP1r18 may be involved in these pathologies and a possible target for treatment of RA and OA.

In conclusion, PPP1r18 and the PP1 complex regulate c-fos dephosphorylation, reduce c-fos nuclear localization, and disturb AP-1 complexation with c-Jun. Thus, PPP1r18 and the PP1 complex repress the transcriptional activity and expression of NFATc1 to subsequently suppress early stage osteoclast differentiation.

## CRediT authorship contribution statement

K. Y. and T. M. performed the experiments. K. Y., T. M., T. S., T. K. and S. K. reviewed the intermediate draft. T. M. and S.K. designed the study and performed the literature review, prepared the initial and final versions of the article. T. M. submitted the document.

## Declaration of competing interest

The authors declare that they have no conflicts of interest.

## Appendix A Supplementary data

Supplementary figures Image 1
