# Supplementary material for: Protein phosphatase 1 regulatory subunit 18 suppresses the transcriptional activity of NFATc1 via regulation of c-fos
Source: Bone Rep. 2021 Aug 4;15:101114. doi: 10.1016/j.bonr.2021.101114 (PMC8353383; doi:10.1016/j.bonr.2021.101114)
Supplement: Supplementary file 1 — Supplementary figures [file mmc1.pdf]

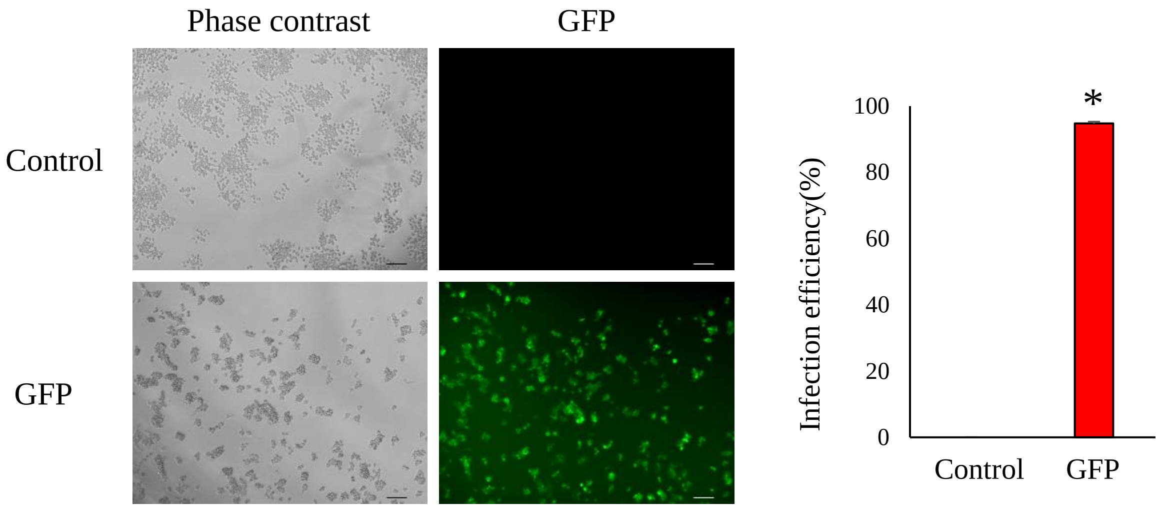

Supplemental figure 1. About 90 % RAW 264.7 cells were introduced DNA by Adenovirus

RAW 264.7 cells were infected empty or GFP adenovirus (M.O.I. = 50) . After 2 days, cells were taken photo (left 4 panels, scale bar; 50 μm). GFP positive cells were counted. (right panel. mean ± SD; n = 4. *, $p < 0.05$.). GFP was expressed in almost 90 % of cells.

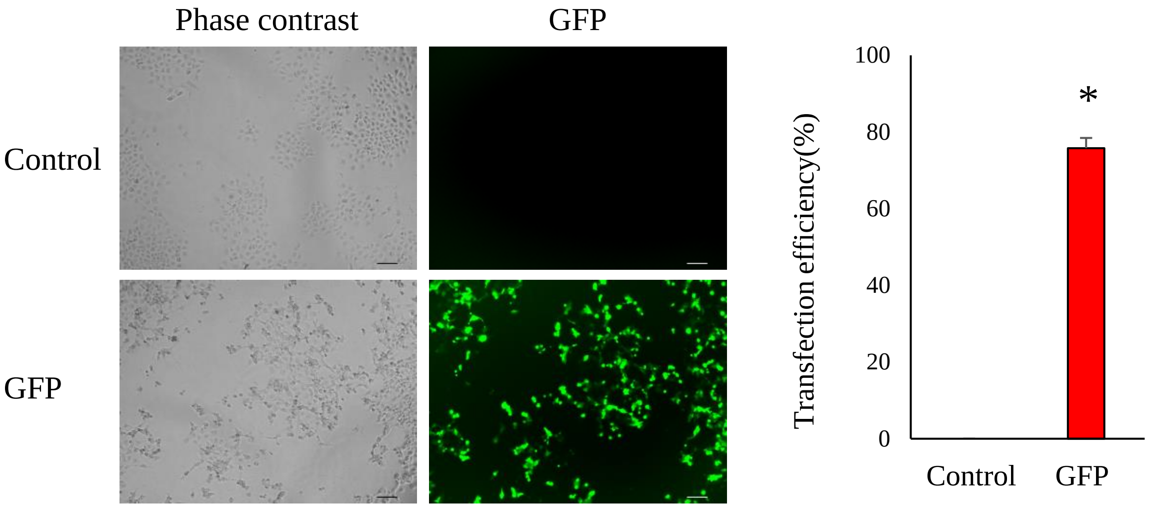

Supplemental figure 2. About 80 % of Cos7 cells are transduced DNA by lipofection

Cos 7 cells was transfected empty or GFP vectors. After 2 days, cells were taken photo (left 4 panels, scale bar; 50 μm). GFP positive cells were counted.(right panel. mean ± SD; n = 4. *, $p < 0.05$.). GFP was expressed in almost 80 % of cells.

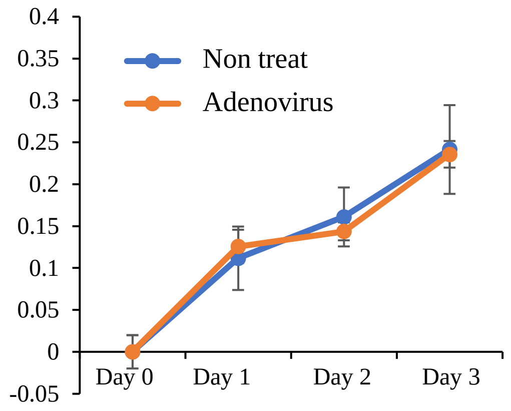

Supplemental figure 3. Adenovirus infection does not affect cell viability and proliferation of RAW 264.7 cells.

RAW 264.7 cells were infected empty adenovirus (M.O.I. = 50) or no treated. Cell survival was determined by Cell counting kit-8 (mean ± SD; n = 3.). There are no significant change by adeno infection.

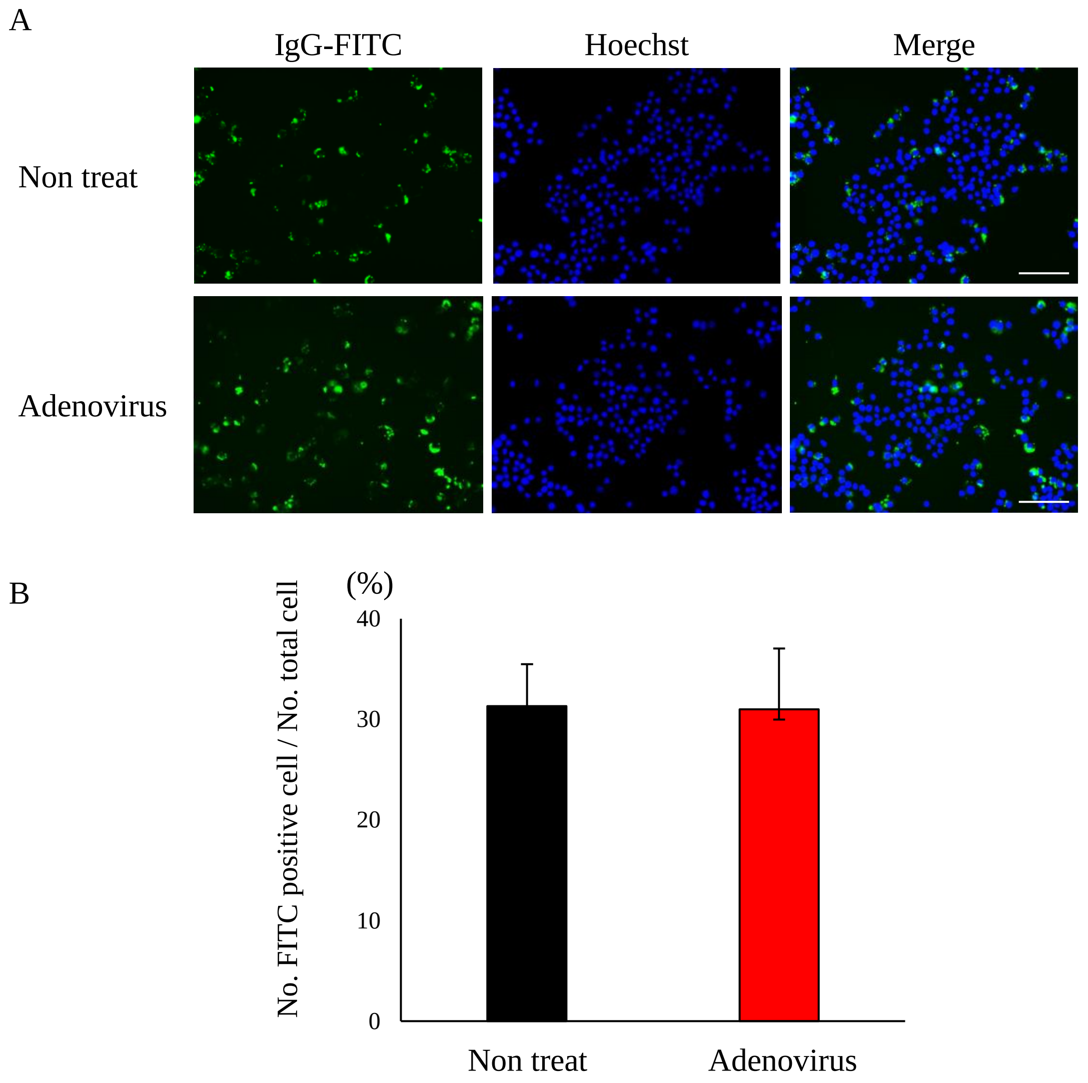

Supplemental figure 4. Adenovirus infection does not affect phagocytosis of RAW 264.7 cells.

RAW 264.7 cells were infected empty adenovirus (M.O.I. = 50) or no treated and cultured for 2 days. Latex beads -rabbit IgG-FITC complex was added (1:200 dilution) in media and incubated for 2 h. After wash, cells were marked by 40 μM of Hoechst 33342 for 10 min. (A) Images were taken by BZ-X810 microscopy. Scale bar, 100 μm. (B) FITC positive cells were counted. (mean ± SD; n = 3). There are no significant change by adeno infection.

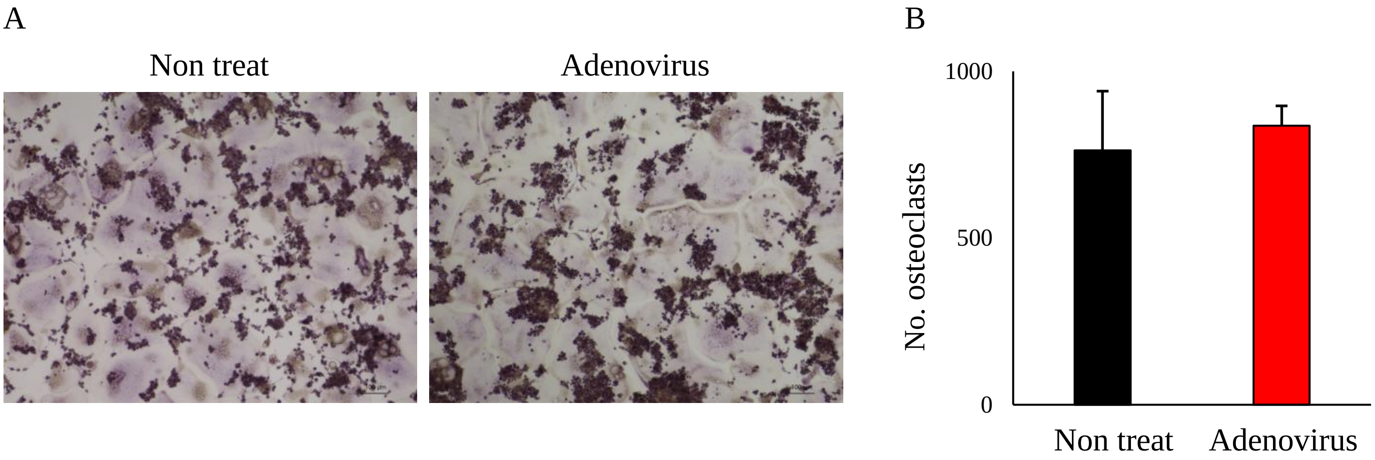

Supplemental figure 5. Adenovirus infection does not affect osteoclast differentiation from RAW 264.7 cells.

RAW 264.7 cells were infected empty adenovirus (M.O.I. = 50) or no treated. After 2 days incubation, cells were differentiated to osteoclasts with 100 ng/ml RANKL. (A) After differentiation, cells were stained by TRAP and images were taken by BZ-X810 microscopy. Scale bar, 100 µm. (B) osteoclasts were counted. (mean ± SD; n = 3). There are no significant change by adeno infection.

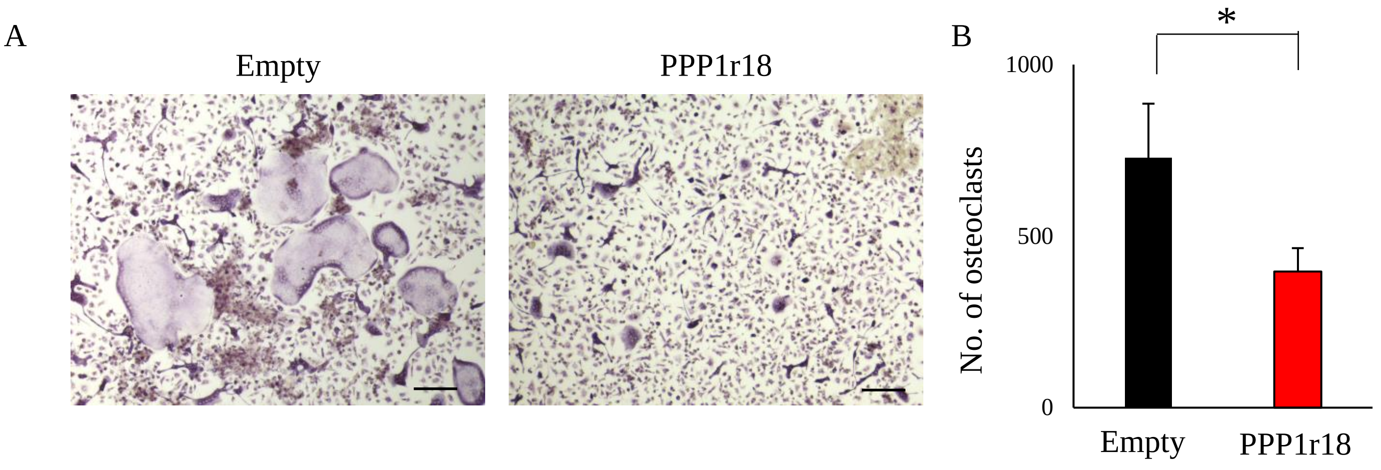

Supplemental Figure 6. PPP1r18 suppresses osteoclast differentiation from bone marrow cells
(A) Empty or PPP1r18 were introduced into bone marrow macrophages by retrovirus. The cells were stimulated with 100 ng/ml sRANKL and 20 ng/ml M-CSF. After 5 days culture, cells were fixed and stained by TRAP staining. Scale bar, 100 μm. (B) Number of TRAP positive multinuclear cells were counted (mean ± SD; n = 3). *, $p < 0.05$.

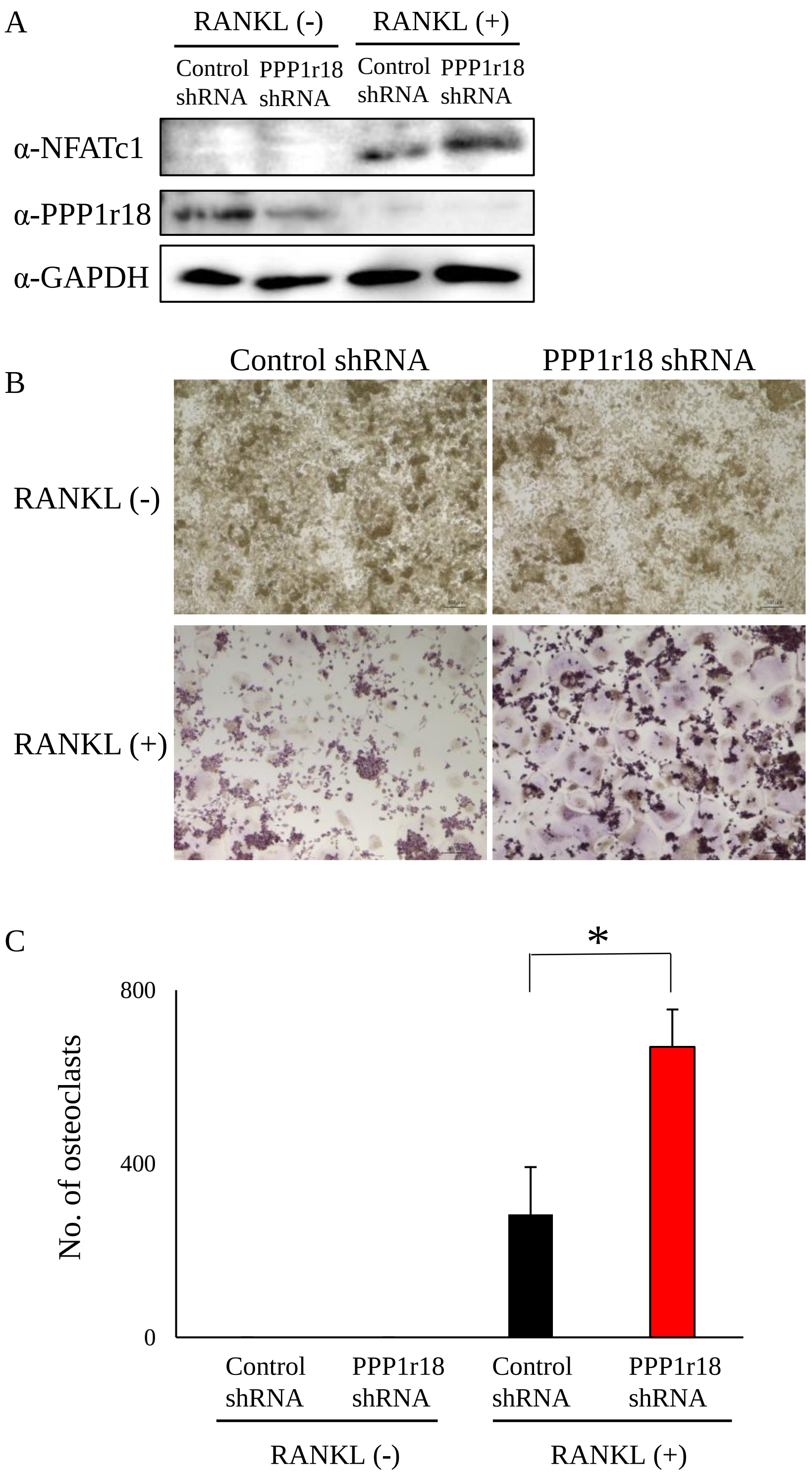

Supplemental Figure 7. suppression of PPP1r18 promotes osteoclast differentiation

Control shRNA or PPP1r18 shRNA adenovirus were infected into RAW 264.7 cells. After 2 days incubation, RAW 264.7 cells were cultured with 100 ng/ml sRANKL for 4 days. (A) Cells were lysed at day 4 and the expression level of NFATc1 and PPP1r18 determined by western blotting analysis. (B) After 4 days culture, cells were fixed and stained by TRAP staining. Scale bar, 100 µm. (C) Number of TRAP positive multinuclear cells were counted (mean ± SD; n = 3). *, $p < 0.05$.

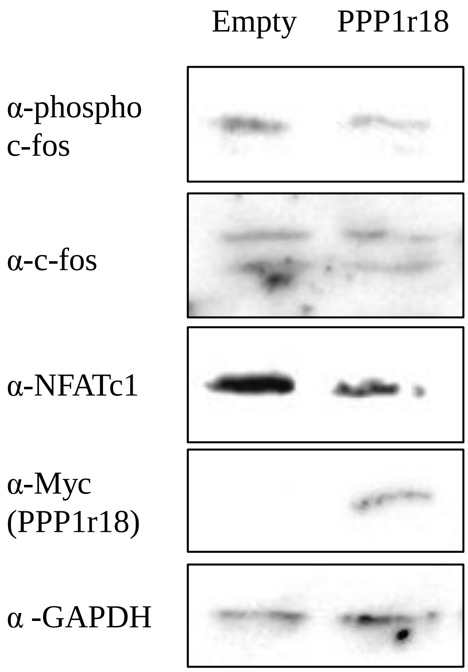

Supplemental Figure 8. c-fos phosphorylation was decreased by PPP1r18 overexpression in BMM
(A) Empty or PPP1r18 were introduced into bone marrow macrophages by retrovirus. The cells were stimulated with 100 ng/ml sRANKL and 20 ng/ml M-CSF. After 3 days culture, cells were lysed and the expression levels of indicated proteins were determined by western blotting analysis.

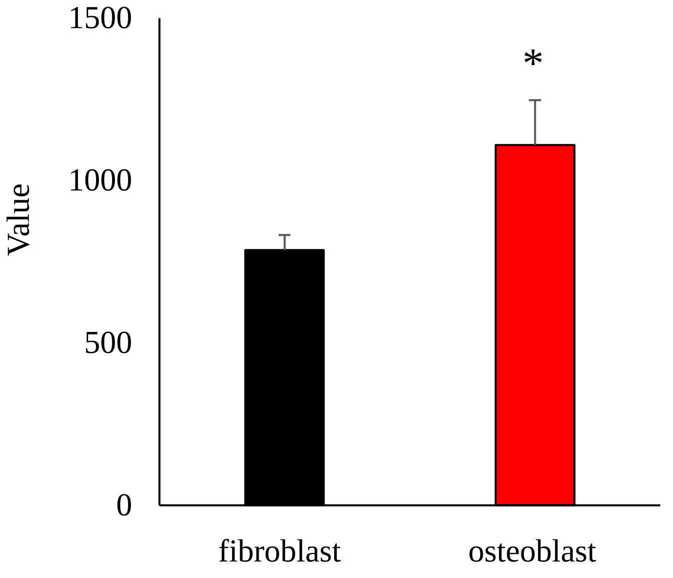

Supplemental Figure 9. PPP1r18 is higher expressed in osteoblast

mRNA expression of PPP1r18 in fibroblast and osteoblast were extracted from GDS2091 dataset (mean ± SD; n=3, *; $p < 0.05$)
